# Associations between serum biomarkers of cartilage metabolism and serum hyaluronic acid, with risk factors, pain categories, and disease severity in knee osteoarthritis: a pilot study

**DOI:** 10.1186/s12891-022-05133-y

**Published:** 2022-03-02

**Authors:** Christos Papaneophytou, Ana Alabajos-Cea, Enrique Viosca-Herrero, Carme Calvis, Marta Costa, Andreas E. Christodoulides, Alexander Kroushovski, Alkis Lapithis, Vaia Maligianni Lapithi, Ioannis Papayiannis, Andreas Christou, Ramon Messeguer, Christoforos Giannaki, Kyriacos Felekkis

**Affiliations:** 1 Department of Life and Health Sciences, School of Sciences and Engineering, University of Nicosia, 2417 Nicosia, Cyprus; 2 Physical Medicine & Rehabilitation Department, Hospital La Fe, 46026 Valencia, Spain; 3 Health Research Institute La Fe, 46026 Valencia, Spain; 4 Drug Development Area, Health & Biomedicine Department, LEITAT Technological Centre, Parc Científic de Barcelona, 08028 Barcelona, Spain; 5 Apollonion Hospital, 2054 Nicosia, Cyprus; 6 Medical School, University of Nicosia, 2408 Nicosia, Cyprus

**Keywords:** Knee osteoarthritis, Biomarkers, COMP, HA, PIICP, OA risk factors, obesity

## Abstract

**Background:**

Specific serum biomarkers of cartilage metabolism such as cartilage oligomeric matrix protein (sCOMP) and procollagen type II C-terminal propeptide (sPIICP) as well as hyaluronan (sHA), a biomarker of synovitis, have been implicated in the pathophysiology of knee osteoarthritis (OA). However, the associations of these biomarkers with the severity of the disease and OA risk factors, including age and obesity remain inconclusive. This analysis examines the associations between these serum biomarkers and the radiographic severity of OA and knee pain, as wells as obesity, the age and gender of the participants, and other OA risk factors.

**Methods:**

From 44 patients with early knee OA and 130 patients with late knee OA we analyzed the radiographic severity of the disease using the Kellgren and Lawrence (KL) grading system. Moreover, 38 overweight healthy individuals were used as a control group. Specific information was collected from all participants during their recruitment. The levels of the three serum biomarkers were quantified using commercially available ELISA kits. Serum biomarkers were analyzed for associations with the average KL scores and pain in both knees, as well as with specific OA risk factors.

**Results:**

The levels of sCOMP were elevated in patients with severe late OA and knee pain and correlated weakly with OA severity. A weakly correlation of sHA levels and OA severity OA was observed. We demonstrated that only sPIICP levels were markedly decreased in patients with late knee OA suggesting the alterations of cartilage metabolism in this arthritic disease. Moreover, we found that sPIICP has the strongest correlation with obesity and the severity of OA, as well as with the knee pain at rest and during walking regardless of the severity of the disease. ROC analysis showed that the area under the ROC curve (AUC) was 0.980 (95% CI: 0.945–0.995; *p* < 0.0001), suggesting high diagnostic accuracy of sPIICP. Interestingly, gender and age had also an effect on the levels of sPIICP.

**Conclusion:**

This study revealed the potential of serum PIICP to be used as a biomarker to monitor the progression of knee OA, however, further studies are warranted to elucidate its clinical implication.

## Introduction

Osteoarthritis (OA) is one of the main causes of pain and disability with knee OA being a leading cause of disability among older adults globally affecting the life quality of patients [1]. The disease is characterized by joint pain and progressive degeneration of articular cartilage involving remodeling of all joint tissues (i.e., bone, synovium, ligaments) and joint space narrowing (JSN) [2, 3]. Therefore, from a physio-pathological perspective, knee OA is a whole joint disease involving structural modifications of the articular cartilage [3] and subchondral bone [4]. Knee OA also involves the disruption of the synovial membrane, meniscus, and the infrapatellar fat pad (IFP) [5]. The estimated prevalence of OA among adults over 60 years of age or older is approximately 13% in women and 10% in men [6]. The major risk factors for developing OA, in addition to age, are gender (female sex), injury, obesity, genetic factors, and high workload [7, 8]. Lifestyle factors including physical activity and exercise have been associated with knee OA, although the evidence is inconsistent [9].

At present, the treatment of OA mainly depends on knee replacement surgery and approaches to reduce symptoms and/or pain [10], while there are no therapies and/or medication approved by regulatory authorities that alter the onset or progression of OA structural damage. The currently available treatments have only moderate effects, and therefore, patients are not satisfied with their efficacy [11]. Due to the failure of the available medications to treat knee OA, the number of joint replacement surgeries is increasing by ~ 10% annually [12]. The anatomical severity of OA is usually assessed by clinical evaluation of pain, joint stiffness, and limitations in physiological function as well as by joint imaging using standard radiographs (X-ray, and magnetic resonance imaging), using the Kellgren and Lawrence scale [13], which is known as the KL grading system (0–4). A KL grade of 0 indicates an intact joint without any features of OA, and the subsequent grades 1–4 represent increasing severity of the disease, and a score > 2 is indicative of OA being present. However, radiography reveals only changes in bone and cartilage, which tend to occur late in the disease [14].

Another way to monitor structural changes in OA is by measuring molecular markers (biomarkers) that are released into the blood and other biological fluids (e.g., urine) during the turnover of tissue [15]. In the past few years, the potential of molecules involved in the bone and cartilage metabolism as biomarkers for knee OA has been investigated (for a review on the topic see [16]). These biomarkers can be determined using commercially available ELISA kits [17]. Biomarkers in osteoarthritis can be categorized using BIPED (Burden of Disease, Investigative, Prognostic, Efficacy of Intervention, and Diagnostic) classification scheme proposed by the Osteoarthritis Biomarkers Network [18] and could be used to detect and monitor bone and cartilage turnover, as well as synovial metabolism for the evaluation of the pathophysiological processes that lead to both joint failure and pain in OA patients [19].

Specific biomarkers have been related to the presence and/or severity of knee OA in cross-sectional studies; whereas longitudinal studies have revealed some markers that could predict OA progression [15]. In the cases of knee OA, a biomarker could be an operator (effector molecule) and/or the result of joint damage. For example, cartilage oligomeric matrix protein (COMP), an important degradation product of articular cartilage is associated with OA severity [20]. COMP is a pentameric non-collagenous glycoprotein primarily identified in cartilage [8, 9], which is a member of the family of thrombospondin and acts as a catalyst in collagen formation [21]. Furthermore, the association of the levels COMP, with the radiographic severity of knee OA has been reported [22]. Also, in the case of cartilage extracellular matrix fragments, hyaluronan (HA) that reflects the extent of synovitis [23], may serve as both a biomarker and stimuli for the innate immune chronic wound healing response in the OA joint [24]. HA is a non-sulfated glycosaminoglycan consisting of alternately repeating D-glucuronic acid and N-acetylglucosamine units. HA is abundantly in articular cartilage and synovial fluid and it is partly responsible for lubrication and viscoelasticity of the synovial [25, 26]. HA also regulates several processes in the articular cartilage, synovial fluid, and subchondral bone (reviewed in [27]). Interestingly, both the concentration and chain length of HA are reduced in synovial fluid in patients with knee OA [26] suggesting that the low viscosity of the synovial fluid may cause the wear-and-pain associated with the disease [28]. Previous studies have also demonstrated that HA level in the circulation is associated with the severity of OA (KL grade) [29], age of patients [29, 30].

Other studies have focused on the synthesis and degradation of type II collagen to identify biochemical markers for OA [7]. Type II collagen is a major structural protein of the cartilage and in combination with other collagen types and non-collagenous proteins, including COMP, forms a tensile network for cartilage [7]. The potential of serum procollagen type II C-terminal propeptide (sPIICP), which is cleaved from cartilage type II procollagen following the release of newly synthesized procollagen into the matrix [15, 31] as a biomarker for monitoring the progression of OA has also been investigated [32]. While a large number of studies have been pursued to identify disease-specific biomarkers for OA, only a very limited number have been identified [24]. A successful biomarker should facilitate the evaluation of disease progression, be easily determined using commercially available assay kits, and may help patients to understand their condition [33].

This work aimed to determine the levels of two biomarkers of cartilage metabolism namely COMP, a biomarker of cartilage degradation, and PIICP, a biomarker of cartilage synthesis as well as hyaluronan, a biomarker of synovitis in serum samples from healthy individuals and patients with different degrees of severity of knee OA. As suggested by other studies, we hypothesized that the concentrations of these biomarkers are correlated with the severity of knee OA and might predict the progression of the disease. Thus, a major objective of this work was to examine the relationship between specific biomarkers in circulation and clinical diagnosis. Combining biochemical markers with other risk factors (i.e., obesity, occupational risk factors, age, gender, etc.), may facilitate better monitoring of the progression of the disease and help in identifying asymptomatic knee OA patients.

## Materials and methods

### Study population and clinical assessment

A total of 38 healthy individuals at high risk of developing OA, 44 patients with early knee OA (EOA), and 130 patients with late (established) knee OA (LOA) were recruited as a random population sample to evaluate the correlation of biomarker levels with the severity of knee OA.

### Healthy subjects and patients with early knee osteoarthritis

A total of 82 individuals have been recruited at Hospital La Fe, Valencia, Spain, from Oct 2018 until Dec 2020. Participants were volunteers attending the hospital for other reasons different from knee issues, mainly for a routine medical checkup. They were informed about the project, and the subjects interested in participating were filtered and classified according to the inclusion /classification criteria (see below). A clinical evaluation consisting of physical examination, patient-based questionnaires, radiographs (X-ray and/or Magnetic Resonance Image-MRI), and collection of blood samples was performed on each subject. After this clinical evaluation, subjects were divided into patients with EOA and healthy subjects. EOA was defined according to the criteria proposed by Luyten [34] which are intended only for research purposes and they are the most precise criteria for the diagnosis of EOA described in the literature. It should be pointed out that the patients have not progressed to advanced OA or any other arthropathy during the implementation of this study.

The inclusion criteria for EOA patients were (i) age ≥ 40 years, (ii) KL grade 0–1, weight-bearing (at least 2 projections: PA fixed flexion and skyline for patellofemoral OA), iii) patient-based questionnaires (see section *2.2* “*Clinical assessment”)*, and (iv) patients should present joint line tenderness or crepitus in the clinical examination. The group of patients with early OA included 44 patients (31 women, 13 men). All patients in this group had chronic daily pain of the knee for at least 6 months.

For the healthy group, the inclusion criteria were (i) age ≥ 40 years; (ii) body mass index (BMI) ≥ 25 kg/m^2^; and (iii) KL grade 0. Healthy subjects (*n* = 38) at high risk of developing knee OA included 23 women and 15 men. Obesity has been associated with the onset and development of OA. For this reason, we chose it as an easily identifiable risk factor for filtering healthy subjects at risk of developing knee OA. None of the healthy individuals had evidence of knee OA assessed by clinical examination, questionnaire, and X-ray and/or MRI films of both knees.

Exclusion criteria were the same for both groups: (i) any cognitive disability that hindered viewing of the audio-visual material; (ii) illiteracy; (iii) comprehension or communication difficulties, (iv) insufficient Spanish language comprehension to follow measurement instructions; (v) presence of any rheumatic, autoimmune or infectious pathology. Individuals with diseases that might increase the levels of sCOMP and/or sHA such as cardiovascular diseases [35] and inflammatory diseases [36] were excluded from this study.

### Patients with established (late) osteoarthritis

A total of 130 patients with late (established) knee OA (LOA) (98 women, 32 men) undergoing knee replacement surgery at Apollonion Hospital, Nicosia, Cyprus, were enrolled in this study from July 2018 until Jan 2020. The minimum criteria for knee replacement therapy (KTR) were significant, prolonged symptoms including intractable pain affecting the quality of life of patients, with supporting clinical and radiological signs [37]. Recruitment of patients was carried out by clinicians and OA was defined according to the American College of Rheumatology criteria (https://www.rheumatology.org/) for the classification and reporting of osteoarthritis of the knee [38]. The inclusion criteria for this group were as follows: age ≥ 50 years, knee pain, radiological evidence (x-ray images) of OA, crepitus audible/ palpable, and stiffness lasting under 30 min. Patients with post-traumatic osteoarthritis, arthritis due to any autoimmune, infective, or inflammatory rheumatological conditions were also excluded from the study. All women were postmenopausal, and all OA patients were without treatment that could interfere with bone metabolism [39]. All patients had chronic daily pain of the knee for at least 6 months.

### Clinical assessment

Demographic and clinical data, including age, weight, height, and clinical symptoms of the knee joints, were recorded. BMI was calculated as weight (kg) divided by height (m)^2^. The Kellgren and Lawrence (K&L) scoring system (0–4) was used to assess the radiographic severity of OA while in both centers the same radiologist, with more than 10 years of experience in musculoskeletal system radiology, examined all the radiographs, and described the KL for each subject [13, 40]. Healthy individuals had a KL grade of 0 in both knees while the patients of the EOA group had a KL score of 0 or 1. Moreover, in this study, patients with established OA were divided into a mild group (KL grade 2 in at least one knee), and a severe group (KL grade 3 or 4 in at least one knee). In this study, because the majority of patients with established OA had both knees affected, the mean KL grade of both knees was used in the analysis (discussed further in “Results”).

All participants were also interviewed regarding pain in both knees by asking: “Have you experienced left or right knee pain in the past months/years, during walking and/or at rest?”. Knee pain at rest and during walking (before surgery for the patients of the late OA group) was assessed using the visual analog scale (VAS*)*, an 11-point (0–10) numerical rating scale (0 = no pain; 10 = worst pain) [41]. All answers were recorded specifically for each knee [answer possibilities were: (1) no; (2) yes, in the right knee; (3) yes, in the left knee; or (4) yes, in both knees]. Individuals were defined as that i) without pain if they indicated a VAS score of zero and ii) with pain if they indicated a score more than zero. For data analysis in this work the average pain in both knees was used and for the graphical depiction of associations with biomarkers knee pain was divided into tertiles resulting in three groups i.e., low (0.5–3.5), moderate (4.0–7.0), and severe (7.5–10).

Patients were asked to participate in this research project with a voluntary decision and they should be competent to understand what is involved and thus, all patients provided written informed consent before study enrolment. A questionnaire was also prepared to collect specific information from each patient during their recruitment while we got approval from the Cyprus National Bioethics Committee (ΕΕΒΚ/ΕΠ/2018/19) and the Ethics Committee on Drug Research of the University and Polytechnic Hospital La Fe, to perform the study. Family history of OA was assessed, as suggested in the literature [42], by the question “Have any of your closest relatives (including children, siblings, parents, and grandparents) had OA?” Response options: “Yes, one individual”, “Yes, two or more individuals”, “No”, or “I do not know”. Both responses “Yes, one individual” and “Yes, two or more individuals” were categorized as having a family history of OA.

Occupational physical exposure (occupational OA risk) was evaluated using the question: “For the job or occupation you had for the longest time, did you do any of the following nearly every day?” with the following answer options: “bending for 2 hours or more”, “walking for 2 hours or more over level ground”, “kneeling for 30 minutes or more”, “squatting for 30 minutes or more”, “climbing a total of 5 or more flights of stairs”, “lifting or moving objects of 10 kg or heavier”, “driving a car for 4 hours or more”, “none of the above”. The occupational risk was included as a categorical variable (yes/no) in statistical analyses. This question has previously been used in [42]. Participants reported if they had had an injury on their knees and/or hips that caused them to visit a doctor, and if they had had previous surgery on their knee(s) and/or hip(s). Participants with a previous knee injury that could not lead to OA, i.e., soft tissue injury without a fracture that could lead to arthritis or at least without a fracture involving the joint surfaces which could lead to arthritis were included in this study.

### Sample collection and determination of biomarkers levels

For the collection of blood, separation of serum, and long storage of the samples we followed the rules proposed by the Standard Operating Procedures Internal Working Group (SOPIWG)/ Early Detection Research Network (EDRN) for specimen collection (including blood samples and management for biomarker discovery and validation) [43]. All samples were stored at 4 °C to prevent hemolysis and processed within 4 h after collection. The samples were maintained at 2–8 °C while handling. Serum samples were stored in 0.5–1.0 mL aliquots, at -80 °C. Also, freeze-thaw cycles of the samples were avoided. All samples used were clear and transparent. Serum samples from healthy individuals and patients with EOA obtained at Hospital La Fe (Valencia, Spain) were stored and processed by the Biobanco La Fe (PT17/0015/0043), following standard operating procedures with the appropriate approval of the Ethics and Scientific Committees.

Serum biomarker levels were determined in the following way: After thawing, serum samples were centrifuged at 2500 g for 10 min at 4 °C and then diluted 1:100 for COMP, 1:8 for HA, 1:4 for PIICP. Determination of COMP and HA levels was carried out using the Human COMP Quantikine ELISA Kit (assay range: 0.2–10 ng/mL) and Hyaluronan Quantikine ELISA Kit (assay range: 0.6–40 ng/mL), respectively from R&D systems (Minneapolis, USA). PIICP levels were determined using the Human Procollagen II C-Terminal ProPeptide (PIICP) CLIA Kit (assay range: 15.63–1000 pg/mL) from Abbexa (Cambridge, United Kingdom).

All ELISA experiments were performed according to the instructions provided by the manufacture of each kit without any modification. All assays employed the quantitative sandwich enzyme immunoassay technique. The concentration of the antigens of interest (i.e., biomarkers) in serum samples was determined using a relative standard curve. Samples were measured in triplicate and the mean values were used in the analysis. The intra- and inter-assay coefficient of variance (CV) were < 10.1 and 12.3% respectively for COMP, < 6.5 and 9.7% respectively for HA and each < 8% for PIICP.

### Statistical analysis

Continuous variables with normal distribution were expressed as means ± standard deviation (SD). Non-normally distributed variables were expressed as medians and interquartile range (IQR). The normal distribution of continuous data was analyzed with the D’Agostino & Pearson omnibus normality test. Significant differences in demographic data that followed Gaussian distribution were calculated using unpaired *t*-test or one-way ANOVA and adjusted by Tukey’s multiple comparison method tests for multiple comparisons. The D’Agostino & Pearson omnibus normality test was used to assess that the levels of biomarkers in serum samples of the total study population, as well as of each of the KL grade groups, were normally distributed, which they were not, and therefore nonparametric tests were subsequently used. Thus, biomarker concentrations between KL grade groups (OA severity) and pain groups (pain at rest and during walking) were compared using the Kruskal-Wallis *H*-test and followed by Dunn’s multiple comparison test. The Mann-Whitney *U-*test was employed to compare the biomarker levels (pairwise comparison) in age groups (i.e., for healthy individuals and patients with EOA: 40–54 years and ≥ 55 years and patients with LOA: 50–69 years and ≥ 70 years) and gender groups. Age grouping for healthy individuals and patients with EOA was carried out taking into account the mean age of participants in these groups, which was approximately 55 years while the mean age of the participants in the LOA was ~ 70 years (Table 1). The linear relationship of serum biomarkers with continuous variables [i.e., age, BMI, KL grade, and pain at rest (resting VAS) or during walking (walking VAS)] was carried out using Spearman’s correlation coefficient. Correlations were classified as very weak (correlation coefficient (*r*) < 0.20), weak (*r* = 0.20–0.39), moderate (*r* = 0.40–0.59), strong (*r* = 0.60–0.79), or very strong (*r* > 0.80). A multiple linear regression analysis was also conducted to evaluate the serum biomarker levels and OA risk factors including age, BMI, KL grade, pain during resting and walking, familial OA, and occupational risk. Finally, ROC curve analysis was used to evaluate the diagnostic value of serum biomarker expression in patients with early or late OA. Other diagnostic parameters were also evaluated, including sensitivity, specificity, cut-off value, and area under the ROC curve (AUC) with a 95% confidence interval (CI), to assess the discrimination power of each biomarker. All reported *p*-values were two-tailed and *p*-values < 0.05 were considered statistically significant. Statistical analysis was performed using GraphPad Prism (v.8.2, GraphPad Software Inc., San Diego, CA, USA). ROC curve analysis and calculation of cut-off point for PIICP were carried out using MedCalc Statistical Software version 19.2.6 (MedCalc Software, Ostend, Belgium).

**Table 1:** Demographic data and characteristics of the subjects in this study

Variable	Healthy	EOA	LOA		
			Total	Mild ^**a**^	Severe ^**b**^
n (%)	38	44	130	35	95
Men	15 (39.5)	13 (29.5)	32 (24.6)	3 (8.6)	29 (30.5)
Women	23 (60.5)	31 (70.5)	98 (75.4)	32 (91.4)	66 (69.5)
Age, years (range)	50.7 ± 6.3^*^ (39–62)	52.4 ± 5.6^#^ (42–65)	70.3 ± 8.3 (50–85)	68.1 ± 8.66 (50–81)	71.1 ± 8.0 (50–85)
BMI, kg/m^2^ (Range)	28.30 ± 2.67 (25.04–35.83)	27.02 ± 4.27 ^§,‡^ (18.62–38.83)	29.98 ± 4.69 (20.78–46.88)	28.73 ± 4.59 (20.90–40.00)	30.44 ± 4.67 (20.78–46.88)
Family history of OA, n (%)					
Yes	26 (68.4)	34 (77.3)	78 (60.0)	25 (71.4)	53 (55.8)
No	12 (31.6)	8 (18.2)	50 (38.5)	10 (28.5)	40 (42.1)
I do not know	0	2 (4.5)	2 (1.5)	0	2 (2.1)
Occupational risk, n (%)	16 (42.1)	25 (56.8)	34 (26.2)	4 (11.4)	30 (31.6)
Previous knee injury, n (%)	15 (39.5)	19 (43.2)	29 (22.3)	10 (28.6)	19 (20.0)
Left Knee					
Pain at rest (0–10)	–	2.3 ± 1.5 (0–8)	4.6 ± 2.3 (0–9)	4.2 ± 2.0 (0–8)	4.8 ± 2.9 (0–9)
Pain at walking (0–10)	–	3.6 ± 2.4 (0–10)	6.0 ± 2.3 (0–10)	5.8 ± 1.9 (1–9)	6.0 ± 2.4 (0–10)
KL grade, n (%)					
0	38 (100)	42 (95.5)	–	–	–
1	–	2 (4.5)	4 (3.1)	0	4 (4.2)
2	–	–	42 (32.3)	35 (100.0)	7 (7.4)
3	–	–	78 (60.0)	–	78 (82.1)
4	–	–	6 (4.6)	–	6 (6.3)
Right Knee					
Pain at rest (0–10)	–	2.9 ± 2.7 (0–8)	4.6 ± 2.2 (0–9)	3.9 ± 2.2 (0–8)	4.8 ± 2.3 (0–9)
Pain at walking (0–10)	–	4.1 ± 1.8 (0–10)	6.0 ± 2.3 (0–10)	5.4 ± 2.6 (0–10)	6.2 ± 2.2 (0–9)
KL grade, n (%)					
0	38 (100)	38 (86.4)	–	–	–
1	–	6 (13.6)	10 (7.7)	1 (2.9)	9 (9.5)
2	–	–	38 (29.2)	34 (97.1)	4 (4.2)
3	–	–	73 (56.2)	–	73 (76.8)
4	–	–	9 (6.9)	–	9 (9.5)

Except where indicated, data are presented as mean ± standard deviation

*BMI* Body mass index, *OA* Osteoarthritis, *EOA* Early OA, *LOA* Late OA, *KL* Kellgren and Lawrence grade

^a^Mild-LOA: KL of 2 in at least one knee; ^b^ Severe-LOA: KL of 3 or 4 in at least one knee

Differences among groups were compared by analysis of variance or Student’s t-test

^*^p < 0.0001: healthy group vs i) Total-LOA, ii) LOA-mild, and iii) LOA-severe OA groups

^#^ *p* < 0.0001: EOA group vs i) LOA, ii) mild, and iii) severe OA groups

^§^ *p* < 0.01: EOA group vs LOA group

^‡^ *p <* 0.001: EOA group vs Severe group

## Results

### Description of the study population

The demographic and clinical characteristics of the study population are illustrated in Table 1. To be included in the analysis, participants had to have a knee radiographical assessment for both knees and have replied to all questions of the questionnaires. The study involved 38 healthy overweight individuals (23 women, 15 men) at high risk of developing OA with a mean age (± SD) of 50.7 ± 6.3 years and a mean BMI (± SD) of 28.30 ± 2.67 kg/m^2^. The early knee-OA group consisted of 44 participants (31 women, 13 men). Mean age (± SD) and mean BMI (± SD) in this group was 52.4 ± 5.6 years and 27.02 ± 4.27 kg/m^2^, respectively. No statistically significant differences in age or BMI were observed between men and women in both the healthy and early OA groups. In this study, 130 patients (32 men and 98 women) with late (established) OA were also recruited. Patients with LOA were also divided into a mild group (KL grade of 2 in at least one knee, *n* = 35), and a severe group (KL grade of 3 or 4 in at least one knee, *n* = 95). Mean age and BMI (± SD) in the total population of patients with LOA were 70.3 ± 8.3 years and 29.98 ± 4.36 kg/m^2^, respectively. No statistically significant differences in age or BMI were observed between men and women in these groups. KL scores ranged from 1 to 4. The majority of the participants (64.6%) had a KL score of ≥3 while all OA patients had both knees affected, and 71 patients (54.6%) had a KL score of ≥3 in both knees. Also, there were no statistically significant differences in KL scores between the left and right knees in these groups. OA was graded KL ≥3 in 26 of 32 (81%) men and 58 of 92 (59.2%) women (*p* > 0.05). There were no statistically significant differences between men and women in pain scores (*p* > 0.05), except the pain score in the right knee (*p* < 0.01). There were also statistical differences in pain scores between patients with early- and late- OA *(p* < 0.01). In addition, there were no statistically significant differences in KL scores between the left and right knees (*p* > 0.05) in all groups as revealed by Student’s *t*-test (data not shown).

### Assessment of prevalence of knee OA according to different definitions

The prevalence of knee OA was assessed based on different definitions among participants per group, and all observations are presented in Table 2. Initially, we assessed the prevalence of knee OA using a 15-scale bilateral definition i.e., a combination of KL grades of both knees (0:0), (1:0), (1:1), …, (4:2), (4:3), (4:4), as illustrated in Table 2. However, according to this definition, some groups consisted of a small number of participants. Therefore, we subsequently, assessed knee OA prevalence using a 9 -scale bilateral definition as previously described [44] i.e., the mean KL grade of both knees to allow linear associations (Table 2). The most prevalent mean KL grade was 0.0 followed by 3.0 and 2.0 accounting for 35.8, 29.7, and 16.0% of participants, respectively. The prevalence of knee OA according to this definition in the total population of this study as well as per group is summarized in Table 2. It should be pointed out that the main advantage of combining KL scores is that smaller group numbers are created (i.e., 15 using the combination of KL scores vs 9 using the bilateral mean KL scores) which enhances the reported confidence intervals [44]. Importantly, the KL grades of the knees are neither omitted (e.g., in several reports the KL grade that is used for analysis is the higher one in patients who have both knees affected) nor summarized [44]. Therefore, in this study, for analysis, the mean KL grades of both knees were used.

**Table 2:** Prevalence of knee OA according to two different definitions

**Prevalence of knee OA based on a combination of KL grades**						
Combination of KL grades	Group, n					Total Population ^a^
	Healthy	Early OA	Late OA			
			Total	Mild	Severe	
0:0	38	38	–	–	–	76
1:0	–	4	–	–	–	4
1:1	–	2	–	–	–	2
2:0	–	–	–	–	–	0
2:1	–	–	1	1	–	1
2:2	–	–	34	34	–	34
3:0	–	–	0	–	–	0
3:1	–	–	10	–	10	10
3:2	–	–	10	–	10	10
3:3	–	–	63	–	63	63
4:0	–	–	0	–	0	0
4:1	–	–	3	–	3	3
4:2	–	–	1	–	1	1
4:3	–	–	4	–	4	4
4:4	–	–	4	–	4	4
Total	38	44	130	35	95	212
**Prevalence of knee OA based on the mean of KL grades**						
Mean of KL grades	Group, n					Total Population ^a^
	Healthy	Early OA	Late OA			
			Total	Mild	Severe	
0.0	38	38	–	–	–	76
0.5	–	4	–	–	–	4
1.0	–	2	–	–	–	2
1.5	–	–	1	1	–	1
2.0	–	–	44	34	10	44
2.5	–	–	13	–	13	13
3.0	–	–	64	–	64	64
3.5	–	–	4	–	4	4
4.0	–	–	4	–	4	4
Total	38	44	130	35	95	212

^a^Total population = health individuals + patients with early OA + total number of patients with late OA

### Correlation of biomarker levels and OA severity

**Fig. 1:**
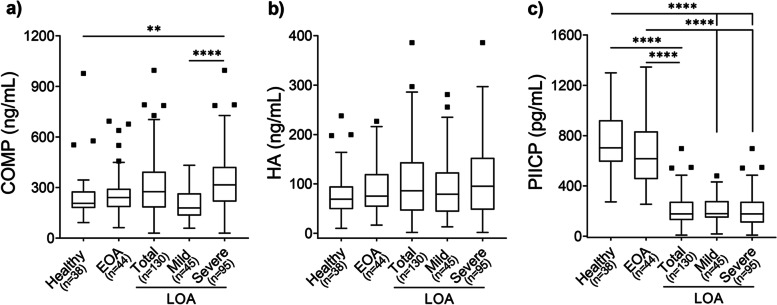
Tukey’s box-and-whisker plots showing the concentration of serum COMP (**a**), HA (**b**), and PIICP (**c**) by OA severity (Kellgren-Lawrence (KL) score). Patients with late osteoarthritis (LOA) were divided into the mild (KL of 2 in at least one knee) and severe (KL of 3 and 4 in at least one knee) groups. Data are presented as medians (IQR). Kruskal Wallis test followed by Dunn’s multiple comparisons test was used for statistical analysis. Only statistically significant differences are shown and indicated with asterisks: ** *p* < 0.01, ****p < 0.0001. The number of participants in each K&L score is shown in parentheses

The levels of the three biomarkers in the healthy group, EOA, and LOA groups as well as in patients with mild-LOA and severe-LOA are illustrated in Fig. 1 (a-c). We initially compared the levels of the three biomarkers among healthy individuals, EOA, and LOA (total population) groups using the Kruskal Wallis and adjusted for multiple comparisons by Dunn’s multiple comparisons method with an α of 0.05. As shown in Fig. 1a and Fig. 1b, respectively, median (IQR) sCOMP and sHA values were higher in the LOA group compared to the EAO and healthy group, but these differences did not reach statistical significance (*p* > 0.05). There were no statistically significant differences in the concentrations of sCOMP, sHA, and sPIICP between the healthy group and the EOA group (Fig. 1 a-c). Interestingly, the sPIICP levels were significantly lower (*p* < 0.0001) in the LOA group compared to those in the healthy and EOA groups as resulted by Kruskal-Wallis test followed by Dunn’s multiple comparisons (Fig. 1c).

We subsequently examined the differences in biomarker levels among healthy individuals, EOA, mild- and severe-LOA groups using the Kruskal Wallis and adjusted for multiple comparisons by Dunn’s multiple comparisons method with an α of 0.05. Based on this classification of LOA, the median (IQR) sCOMP levels of 316.0 (217.2–421.88) ng/mL in the severe-LOA group were significantly higher compared to those in the healthy [206.6 (178.9–277.3) ng/mL, *p* < 0.01] and the EOA group [178.8 (134.0–265.4) ng/mL, *p* < 0.0001] (Fig. 1a).

However, there was no statistically significant difference (*p* > 0.05) in the sCOMP levels between the EOA group and i) healthy group and ii) severe-LOA group. Serum HA levels showed a trend of slight increase as OA severity increased, but these differences did not reach statistical significance (*p* > 0.05) (Fig. 1b). On the contrary, the sPIICP levels showed a trend of decreased values as OA severity increased (Fig. 1c). Median values (IQR) of sPIICP levels [703.4 (593.0–922.8) pg/mL] were significantly (*p* < 0.0001) higher in the healthy group compared to the mild-LOA group [179.1 (148.4–278.9) pg/mL] and severe-LOA group [177.6 (109.1–273.3) pg/mL]. There was also statistically significant difference (*p* < 0.0001) in sPIICP levels between the EOA group and the mild- LOA group as well as the severe-LOA group. There was no statistically significant difference (*p* > 0.05) in sPIICP levels between the health and EOA groups as well as between the mild and severe OA groups (Fig. 1c).

Spearman correlation was also employed to assess the collinearity of the biomarkers (Table 3). The results revealed that sCOMP correlated very weakly and negatively with sPIICP in the total population (*r* = − 0.184, *p* < 0.01). In the healthy group, there were no correlations among the three biomarkers. Interestingly, in the EOA group, sCOMP was positively and moderately correlated with sPIICP (*r* = 0.448, *p* < 0.01). In the LOA group, sCOMP was very weakly and positively correlated with sHA (*r* = 0.177, *p* < 0.05) while a weak and negative correlation was obtained between sCOMP and PIICP (*r* = − 0.307, *p* < 0.01).

**Table 3:** Correlation between biomarker levels in the total population, healthy individuals, and patients with EOA or LOA

Biomarkers	Total population		Healthy		EOA		LOA	
	*r*	*p*	*r*	*p*	*r*	*p*	*r*	*p*
COMP vs HA	0.121	> 0.05	0.072	> 0.05	0.017	> 0.05	**0.177**	**< 0.05**
COMP vs PIICP	**−0.184**	**< 0.01**	0.086	> 0.05	**0.448**	**< 0.01**	**−0.307**	**< 0.0001**
PIICP vs HA	−0.030	> 0.05	0.050	> 0.05	−0.117	> 0.05	0.123	> 0.05

*EOA* Early Osteoarthritis, *LOA* Late Osteoarthritis. Correlation analysis between biomarker levels was performed using Spearman’s rank correlation coefficient (r). All reported *p*-values are two-tailed. *p-v*alues < 0.05 were considered statistically significant and indicated in bold

### Correlation between biomarker levels and knee pain

**Fig. 2:**
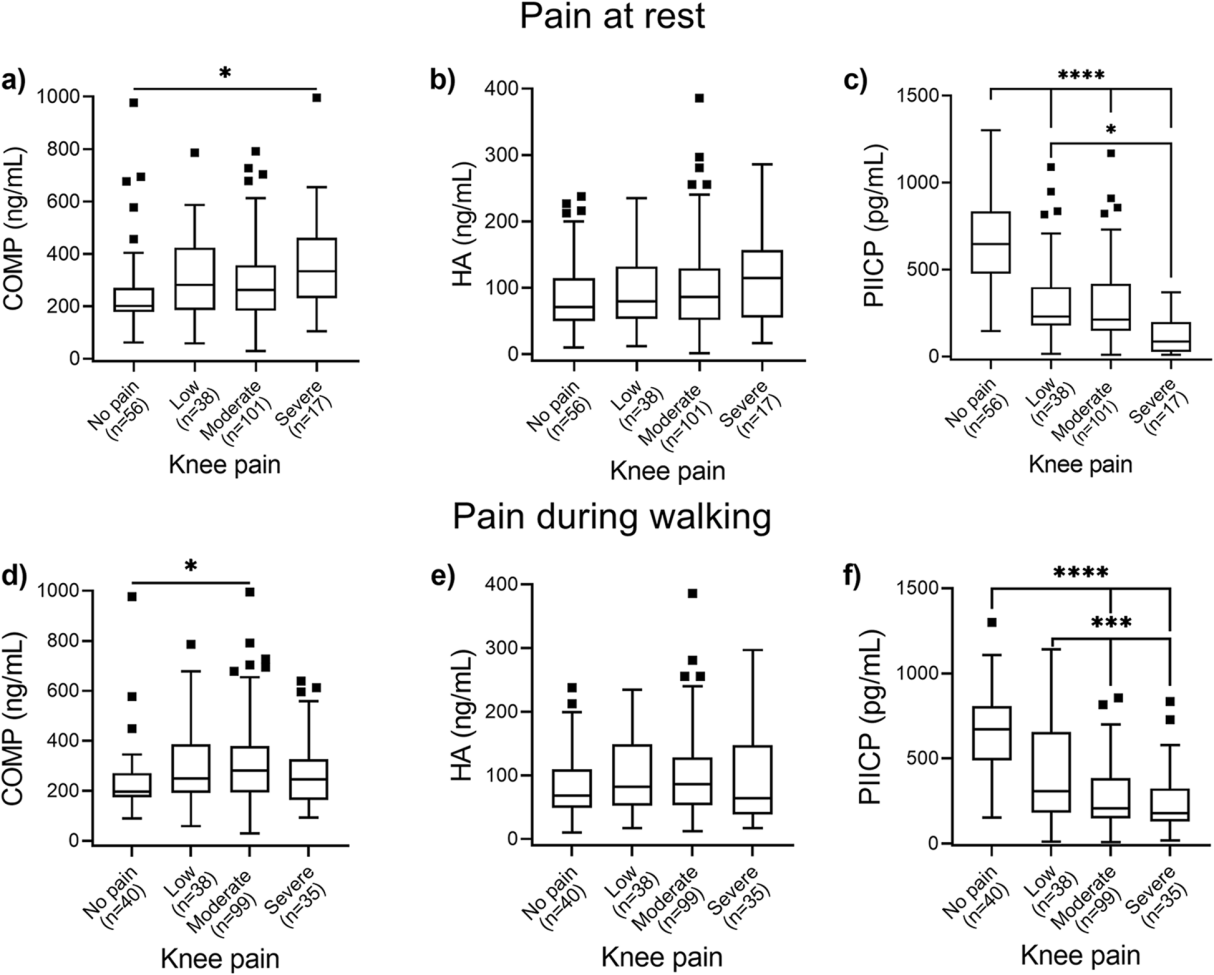
Tukey’s box-and-whisker plots showing the biomarker levels according to knee pain severity at rest (**a-c**) and during walking (**d-f**). Data are presented as medians (IQR). Kruskal Wallis test followed by Dunn’s multiple comparisons test was used for statistical analysis. Only statistically significant differences are shown and indicated with asterisks: * *p* < 0.05, ***p < 0.001, ****p < 0.0001

Patients were divided into four groups according to the bilateral mean knee pain intensity both at rest (Fig. 2 a-c) and during walking (Fig. 2 d-f). The presence of low or moderate pain did not affect the levels of sCOMP, however, the levels of this biomarker were significantly higher (*p* < 0.05) in the group with severe pain compared to those without knee pain at rest (Fig. 2a). Also, the levels of sCOMP were higher in the group with moderate pain during walking (*p* < 0.05) compared to the group without pain (Fig. 2d). There was no significant difference (*p* > 0.05) in sHA levels among the four pain groups both at rest (Fig. 2b) and during walking (Fig. 2e). The levels of sPIICP were significantly higher in individuals without knee pain at rest compared to those with low, moderate, and severe knee pain (*p* < 0.0001) (Fig. 2c). There was also statistically significant difference in sPIICP levels between the low- and severe-pain at rest groups (*p* < 0.05). There was statistically significant difference in the sPIICP levels between the group without pain during walking and i) the group with moderate (*p* < 0.0001) and ii) severe pain group (*p* < 0.0001). There was also statistically significant difference in sPIICP levels between the group with low pain during walking and both the moderate and severe pain groups (*p* < 0.001) (Fig. 2f).

### Correlation of biomarker levels and clinical parameters

Spearman’s correlation coefficient was calculated to examine the association between the levels of biomarkers in serum and risk factors (continuous factors) for OA including age, BMI, KL grade (bilateral mean value), as well as pain at rest (resting VAS), and walking (walking VAS) (Table 4). The levels of sCOMP increased with age and BMI and were weakly correlated with KL grade. Furthermore, sCOMP levels were very weakly correlated with pain at rest, but they were not correlated with pain during walking. Serum HA levels were weakly correlated only with age and very weakly correlated with KL grade. Interestingly, sPIICP levels were significantly and negatively correlated to OA risk factors such as age and BMI, as well as with KL grade and bilateral mean pain intensity at rest and during walking (Table 4). It should be noted that the negative coefficient values indicated that the variables are inversely related (i.e., as the value of one variable increases, the value of the other tends to decrease).

**Table 4:** Association of biomarker levels with age, BMI, KL grade, and knee pain

Variable	Biomarker					
	sCOMP		sHA		sPIICP	
	r	*p*-value	r	*p*-value	r	*p*-value
Age	0.2574	**0.0002**	0.2523	**0.0002**	−0.5807	**< 0.0001**
BMI	0.2150	**0.0016**	−0.0921	0.1818	−0.2225	**0.0011**
KL grade	0.2417	**0.0004**	0.1399	**0.0419**	−0.6776	**< 0.0001**
Resting VAS	0.1607	**0.0192**	0.0263	0.7029	−0.4442	**< 0.0001**
Walking VAS	0.0624	0.3657	0.0059	0.9314	−0.4184	**< 0.0001**

Correlation analysis between biomarker levels and the variables was performed using Spearman’s rank correlation coefficient (r). All reported *p*-values are two-tailed. *p-v*alues < 0.05 were considered statistically significant and indicated in bold

Moreover, we performed a multiple linear regression analysis to identify potential associations among the parameters of age, BMI, pain, KL grade, gender, familiar OA, and occupational risk with each of the biomarkers (Table 5). The results revealed that serum COMP and HA were associated with age and occupational risk, whereas BMI, gender, KL score, pain at resting and during walking as well as with a previous knee injury and familial OA exerted no statistically significant influence on the levels of serum COMP and HA. Interestingly, the levels of serum PIICP were independently associated with KL grades (*p* < 0.0001), while the other factors did not have a statistical relationship with the levels of this biomarker.

**Table 5:** Multiple linear regression for the assessment of OA risk factors on the levels of the three serum biomarkers

Variable	sCOMP^a^				sHA^b^				sPIICP^c^			
	Est	SE	t	*p*-value	Est	SE	t	*p*-value	Est	SE	t	*p*-value
(Constant)	−14.73	103.4	0.143	0.8868	35.95	42.51	0.8458	0.3987	695.4	136.2	5.106	**< 0.0001**
Age (per year)	2.865	1.421	2.017	**0.0450**	1.773	0.584	3.035	**0.0027**	−0.622	1.872	0.3323	0.7400
BMI (per 1 kg/m^2^)	4.191	2.502	1.675	0.0955	− 1.663	1.029	1.616	0.1077	1.228	3.297	0.3725	0.7099
Gender	−30.76	26.32	1.168	0.2440	−4.817	10.83	0.445	0.6569	−62.96	34.69	1.815	0.0710
KL grade	15.08	14.36	1.050	0.2950	2.124	5.907	0.359	0.7195	− 163.5	18.92	8.639	**< 0.0001**
Resting VAS	0.843	6.409	0.132	0.8955	−2.250	2.636	0.8535	0.3944	0.321	8.445	0.0381	0.9697
Walking VAS	−7.532	5.840	1.290	0.1986	−0.615	2.402	0.2561	0.7981	2.459	7.695	0.3195	0.7496
Previous knee Injury	20.49	24.21	0.846	0.3984	5.480	9.956	0.5504	0.5826	6.553	31.90	0.2054	0.8374
Occupational Risk	78.31	25.64	3.054	**0.0026**	21.30	10.54	2.020	**0.0447**	26.83	33.78	0.7941	0.4281
Familial OA	1.902	22.94	0.083	0.9340	−2.966	9.436	0.3144	0.7536	−10.28	30.23	0.3402	0.7341

*p*-values < 0.05 are consider significant and indicated in bold

^a^ R^2^ = 0.153, model ANOVA: F = 4.05, *p* < 0.0001

^b^ R^2^ = 0.112, model ANOVA: F = 2.84, *p* < 0.001

^c^R^2^ = 0.560, model ANOVA: F = 26.81, *p* < 0.0001

**Fig. 3:**
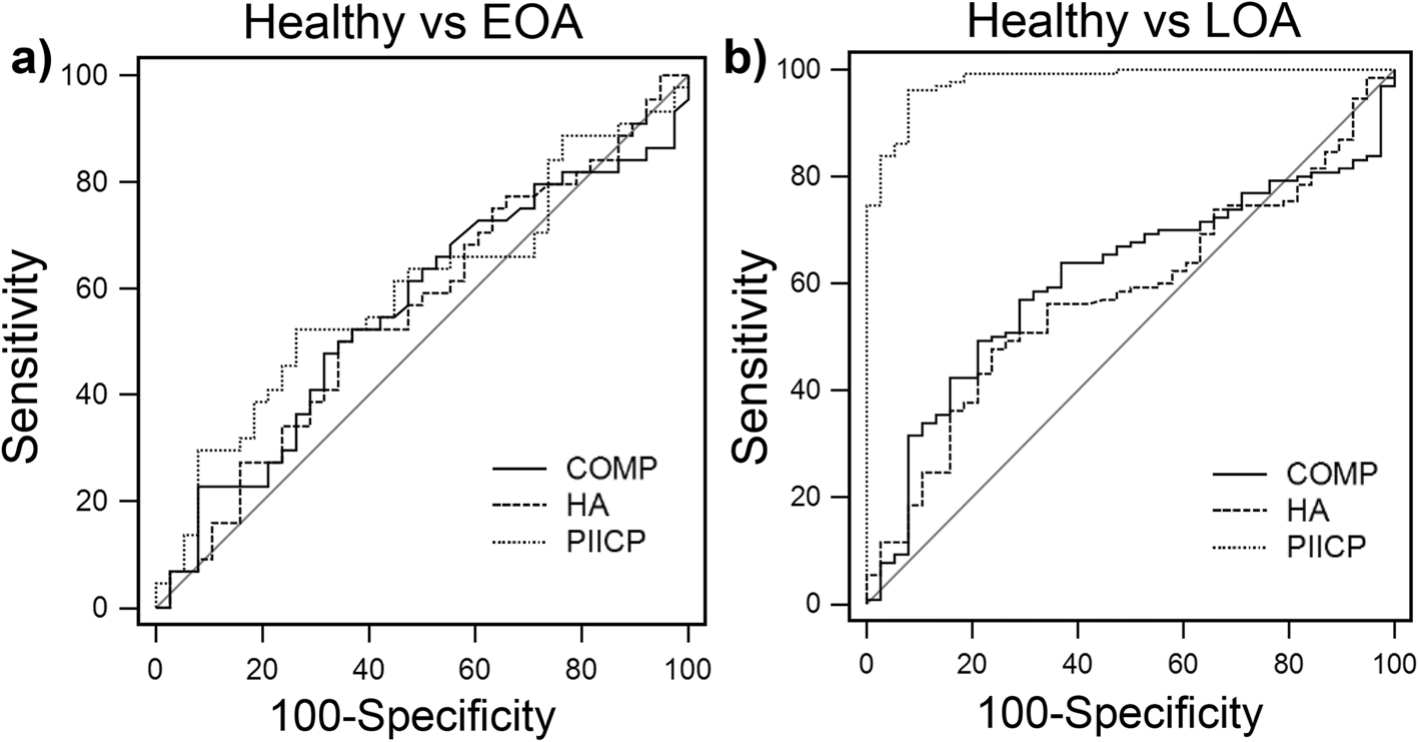
ROC curves for the three individual biomarkers (COMP, HA, and PIIICP) in healthy individuals and patients with early (**a**) and late (**b**) osteoarthritis

The diagnostic efficiency of the three biomarkers i.e., their ability to differentiate patients with EOA or LOA from healthy individuals, was further evaluated by the sensitivity, specificity, Youden index, and the area under the ROC curve (Table 6). The three serum biomarkers have limited diagnostic potential in early knee osteoarthritis because the reliability of the cutoff values for the development of EOA was low (AUC < 0.6; Table 6). The ROC curves for the three serum biomarkers for EOA and LOA are illustrated in Fig. 3.

**Table 6:** RoC Curve Analysis for the diagnosis of patients with EOA or LOA

Parameter	EOA			LOA		
	COMP	HA	PIICP	COMP	HA	PIICP
Sensitivity	47.7	50.0	52.7	49.2	47.5	96.2
Specificity	68.4	65.8	73.7	78.9	76.3	92.1
Youden Index	0.162	0.158	0.2596	0.282	0.240	0.8826
AUC	0.558	0.555	0.596	0.609	0.578	0.980
*p*-value	> 0.05	> 0.05	> 0.05	< 0.05	> 0.05	< 0.0001
95% CI	0.444–0.668	0.441–0.665	0.482–0.703	0.531–0.684	0.500–0.654	0.945–0.995

*EOA* Early Osteoarthritis, *LOA* Late Osteoarthritis, *AUC* Area Under Curve, *CI* Confidence interval

Interestingly, our analysis revealed that among three serum markers, PIICP had the highest AUC value (0.980, 95%CI: 0.945–0.995; *p* < 0.0001) for LOA diagnosis, followed by COMP (Table 6). Therefore, according to our analysis, sPIICP could discriminate between patients with LOA and healthy individuals, with a sensitivity of 92.6% and specificity of 92.1%. The cut-off value of PIICP for LOA diagnosis was 465.4 pg/mL.

### Subgroup analysis

**Fig. 4:**
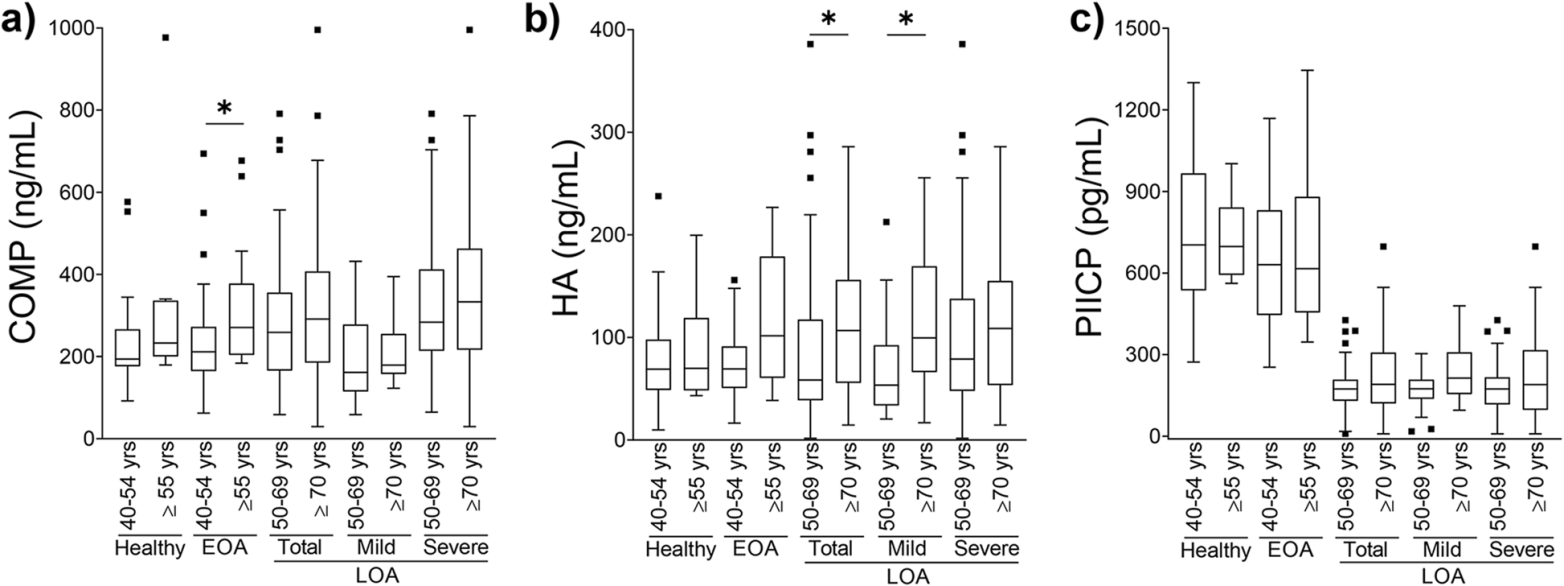
Tukey’s box-and-whisker plots showing the differences in the levels of serum COMP (**a**), HA (**b**), and PIICP (**c**) between the age 40–54 years and age ≥ 55 years groups in healthy participants and early osteoarthritis (EOA) patients and the age 50–69 years and age ≥ 70 years groups in patients with late osteoarthritis (LOA). Patients with LOA were also divided into the mild (KL of 2 in at least one knee) and severe (KL of 3 or 4 in at least one knee) groups. Data are presented as medians (IQR). Mann-Whitney U test was used for pairwise statistical analysis. Only statistically significant differences are shown and indicated with asterisks: * *p <* 0.05

We subsequently divided the participants of the healthy group and EOA into 2 age categories namely age 40–54 years (*n* = 28 in both groups) and age ≥ 55 years groups (*n* = 10 in healthy group and *n* = 16 in EOA group). Likewise, we divided the participants with established (late) OA into two age categories namely age 50–69 years (*n* = 57) and age ≥ 70 years (*n* = 73) groups. Patients with mild- and severe- LOA were also divided into the 50–69 years (*n* = 57) and age ≥ 70 years (*n* = 73) groups (Fig. 4 a-c).

As illustrated in Fig. 4a, in patients with EOA, sCOMP levels were significantly lower (*p* < 0.05) in the age 40–54 years group compared to the age ≥ 55 years group. Median (IQR) values of sHA were significantly higher (*p* < 0.05) in the age ≥ 70 years group compared to the age 50–69 years group in both the total population of patients with LOA as wells as in patients with mild-LOA (Fig. 4b). There was no statistically significant difference in sPIICP levels among all groups (Fig. 4c).

Spearman correlation analysis was then used to assess the correlation between serum biomarkers and the age of participants in the different groups (Table 7). In the healthy and EOA groups, there was a weak correlation between sCOMP levels and the age of participants. In the total population of patients with LOA, there was a weak correlation between the levels of sCOMP and sHA and the age of participants. Interestingly, in the mild-LOA group, there was a moderate correlation between the sHA levels and the age of patients, while in the severe-LOA group a weak correlation between the sCOMP levels and the age of participants was observed.

**Table 7:** Correlation of biomarkers with the age of healthy individuals and patients with EOA and LOA

Biomarkers	Healthy		EOA		LOA					
					Total		Mild		Severe	
	*r*	*p*	*r*	*p*	*r*	*p*	*r*	*p*	*r*	*p*
COMP	**0.349**	**< 0.05**	**0.379**	**< 0.05**	**0.218**	**< 0.05**	0.187	> 0.05	**0.206**	**< 0.05**
HA	0.230	> 0.05	0.295	> 0.05	**0.268**	**< 0.01**	**0.427**	**< 0.05**	0.187	> 0.05
PIICP	0.026	> 0.05	0.122	> 0.05	0.058	> 0.05	0.138	> 0.05	−0.012	> 0.05

*EOA* Early Osteoarthritis, *LOA* Late Osteoarthritis

Correlation analysis between biomarker levels and age of the participants was performed using Spearman’s rank correlation coefficient (r). All reported *p*-values are two-tailed. *p*-values < 0.05 were considered statistically significant and indicated in bold

**Fig. 5:**
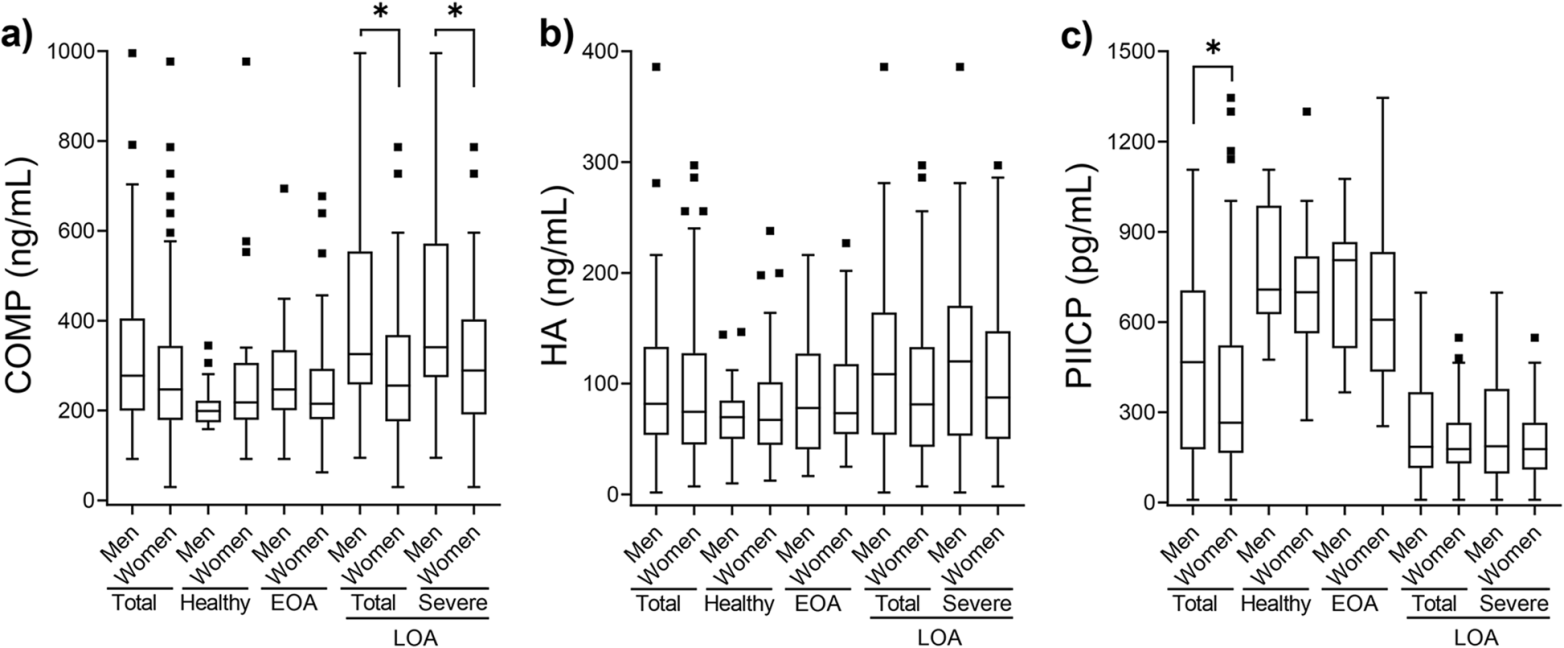
Tukey’s box-and-whisker plots showing the differences in the levels of serum COMP (**a**), HA (**b**), and PIICP (**c**) between men and women in the total population as well as in the healthy group and the groups of early osteoarthritis (EOA) and late osteoarthritis (LOA). Patients with LOA were divided into the mild (KL of 2 in at least one knee) and severe (KL of 3 or 4 in at least one knee) groups. Data are presented as medians (IQR). Mann-Whitney U test was used for pairwise statistical analysis. Only statistically significant differences are shown and indicated with asterisks: * *p <* 0.05

Comparison between genders (Fig. 5) by the Mann-Whitney *U* test revealed that the levels of sCOMP were significantly higher in men compared to women in the total population of patients with LOA (mild and severe) as well as in the severe-LOA group (Fig. 5a). There was no statistically significant difference in sHA levels between men and women in all groups as shown in Fig. 5b. The levels of sPIICP were significantly lower (*p* < 0.05) in females compared to males in the total population of participants (Fig. 5c). It should be noted that statistical analysis of the mild-LOA group was not performed because of the small number of male participants in this group.

## Discussion

This study aimed to examine whether there are differences in the levels of two cartilage biomarkers (COMP and PIICP) and a synovitis biomarker (HA) among overweight healthy individuals, patients with EOA, and patients with LOA and examined whether the levels of these biomarkers in the three groups are associated with OA severity, knee pain at rest and during walking as well as with other OA risk factors. It has been generally accepted that a KL grade of 2 is the cut-off for defining radiographic knee OA features [45]. Therefore, in this study, we divided the patients with established OA into the mild group (KL grade of 2 in at least one knee) and severe group (KL grade of 3 or 4 in at least one knee). Because the majority of the patients in the LOA group had both knees affected, we used a 9-scale bilateral definition, i.e., the mean KL grade of both knees, to assess the severity of knee OA (Table 2).

In the current study, we demonstrate a clear correlation between the markers of cartilage synthesis (PIICP) and the severity of OA. Importantly, ROC analysis revealed that the AUC was 0.980, (95% CI: 0.945–0.995; *p* < 0.001), indicating high diagnostic accuracy of sPIICP i.e., this biomarker is suitable for the differentiation of healthy individuals at high risk of developing OA from patents with LOA (Fig. 3 and Table 6). It should be pointed out that our analysis revealed that sPIICP is not a suitable biomarker for the diagnosis of EOA. Moreover, our results revealed that sPIICP and sCOMP can detect pain-associated differences in patients regardless of the OA severity. The most significant finding of this work was that reduced serum levels of PIICP, which is one biomarker of cartilage synthesis, in patients with established OA were correlated with knee OA risk factors such as age and obesity. Type II collagen is a main component of the cartilage matrix that is synthesized by chondrocytes and its synthesis and breakdown are linked to cartilage metabolism. In detail, type II collagen is synthesized as a premature protein consists of three extra domains: a signal peptide, the procollagen type II N-terminal propeptide (PIINP), and the procollagen type II C-terminal propeptide (PIICP). These propeptides are cleaved off and released into biological fluids (e.g., blood, urine) during maturation [46]. Thus, the levels of these peptides in biological fluids reflect type II collagen synthesis and these two components could be used as markers to monitor the rate of OA progression [15]. It has also been demonstrated that PIICP levels in the joint fluid might be a prognostic marker for early OA in the knee as the concentration of PIICP was found to correlate with risk factors including obesity and varus knee alignment [15]. Post-traumatic damage of cartilage and periarticular tissues may also contribute to the development of OA [47] while obesity has been identified as a risk factor for the development of OA [48]. Our analysis also revealed that sPIICP concentration is also correlated with obesity and associated with occupational risk. In patients with severe OA, we found decreased levels of sPIICP compared to healthy individuals and patients with early OA and these findings are well in agreement with previous studies [31]. Another interesting result in this study was that the alterations in sPIICP levels are associated not only with the severity of knee OA but also with knee pain (Table 4). The mean levels of sPIICP in the groups with mild OA and severe OA were more than 2.5-fold lower than those in the healthy and EOA groups (Fig. 1c) suggesting that this decrease was not solely driven by factors such as age and obesity that are also correlated with the PIICP levels (Table 3). Our results are in good agreement with previous studies suggesting that PIICP concentrations in biological fluids might be an ideal prognostic marker for OA in the knee as the level of PIICP was found to correlate with risk factors such as obesity [7]. The progression of joint damage in OA is likely to result from an imbalance between cartilage degradation and synthesis processes and it has been proposed that quantifying the procollagen peptides in biological fluids could result in a better understanding of OA disease pathology and would provide means for evaluation of anabolic disease-modifying OA drugs [46]. Furthermore, because PIICP is released only during the synthesis of the new molecules, its production is known to reflect the rate of type II collagen synthesis and cartilage metabolism [49]. It was previously reported that both synovial fluid and serum levels of PIICP were increased in individuals with uncertain knee OA (KL grade of 0 or 1) [32]. On the contrary, the serum levels of PIICP were decreased in patients with early-stage of knee OA (K/L grade 2), where the radiographic joint space narrowing became clear [32]. A decrease of sPIICP levels [19, 31] and changes in the ratios of cartilage collagen degradation (C-terminal neopeptide/C2C and telopeptide fragment of collagen type-II/CTX-II) and synthesis (PIICP) markers [15, 19, 50] were also reported with the onset of knee OA (KL grade 2 vs KL grade 1).

Interestingly, we also found a weak correlation between sCOMP levels and OA severity (KL grade), age, and BMI (Table 4). Regression analysis also revealed a correlation between sCOMP levels and i) age and ii) occupational risk (Table 5). COMP is a pentameric non-collagen protein related to the thrombospondin family, and also a constituent of articular cartilage and several studies have demonstrated that sCOMP is elevated in OA and after knee injury [51, 52] and therefore it might have some value as a diagnostic and/or prognostic marker of knee OA [52, 53]. The majority of the previous studies investigating sCOMP as a biomarker for knee OA have shown that the levels of this biomarker are associated with structural and metabolic changes in OA [54]. Some studies investigating the association of sCOMP levels with clinical symptoms have shown inconsistent results [55]. Our analysis revealed that the level of COMP in serum is significantly higher in patients with severe OA (KL grade of 3 or 4) compare to that in the healthy group. Interestingly, our results also demonstrated that the serum level of COMP is also significantly higher in the severe OA group compared to that in the mild OA group, highlighting its potential as a prognostic biomarker as well as a biomarker for monitoring the progression of the disease. Elevated levels of sCOMP have been associated with knee pain [56]. Interestingly it has also been demonstrated that serum COMP levels are increased in patients with pain in the knees and without any radiological abnormalities, indicating early cartilage damage in these patients compared with healthy individuals [57]. In another study, the concertation of COMP in serum has been correlated with pain score but not OA severity (radiological grading), while it also negatively correlated with the progression of the disease. Thus, COMP levels can also be used as a prognostic marker to predict patients at risk of rapid progression [20].

In contrast to previous findings [19, 45, 58], we could not demonstrate significant associations between sHA levels and clinical parameters including KL grade (*r* = 0.1399) and pain at rest (*r* = 0.0263) and during walking (*r* = 0.0059) (Table 4). However, in the multiple regression analysis sHA was correlated only with the KL grade (Table 5). Synovitis plays a vital role in the onset of OA [59] through the production of HA and pro-inflammatory cytokines including tumor necrosis factor-α and interleukin 1β. These cytokines induce the production of matrix metalloproteinase by fibroblasts resulting in the degradation of the articular cartilage matrix [60]. Previous studies have studied the association of sHA with the progression of knee OA [19, 61–63] (see also [45] and references cited therein), however, the data were limited. Sasaki et al. reported the correlation of sHA and radiographic progression of knee OA in a more general population including healthy individuals and patients with both early- and severe- knee OA for 5 years [45]. Even though the levels of sHA were positively correlated with KL grade progression the patients included in the study by Sasaki et al. were only middle-aged women and young individuals at very low risk of developing knee OA [45]. To better evaluate the role of HA in the development and progression of knee OA a wider range of ages may be needed. It has been previously reported that the sHA concentration might be also a specific biomarker for OA of other joints including the lumbar spine [45]. It has also been reported by Inoue et al. [64] that the correlation between sHA levels and OA was higher in patients with knee OA compared to those with hip OA or hand OA. However, sHA levels were increased with age because of an impaired ability of older individuals to metabolize HA [65]. Furthermore, other factors may contribute to the elevated sHA concentration in the elderly population such as hepatic failure, renal failure, and rheumatoid arthritis (RA) [66]. Therefore, patients suffering from hepatic and renal failure as well as from RA were excluded from this study allowing us to evaluate the correlation of sHA concentration and severity of knee OA more accurately. The lack of significant associations between HA levels and OA stage/severity suggests that this biomarker may not be a sensitive measure of the role of bone turnover in the progress of OA. Taken together the result of this study and those of others [33] indicate that sHA levels might not be an ideal biomarker for monitoring the progression of OA in elderly people.

Our analysis also revealed that there were no statistically significant differences in sCOMP and sHA levels between men and women in the total population; however, the sPIICP levels were statistically different (*p* < 0.05). Moreover, biomarker levels between males and female groups of identical OA severity (KL scores) revealed that the levels of sCOMP and sHA levels were higher in females compared to those in males in some but not all KL score groups, but the levels of sPIICP were found to be higher in males compared to females in the majority of KL grade groups suggesting the association of these markers with the female gender in OA [67–69], and also warrants further research on the potential relevance of gender as an isolated factor for the use of these biomarkers in OA in the context of structural features. Females are at a higher risk of knee OA while several epidemiological studies revealed that the prevalence of knee OA in women is higher than in men [6].

### Limitations

This study has some limitations. The recruitment of individuals has been carried out in two different research centers in two different countries (Spain and Cyprus) and thus we compared cohorts that differ not only in knee OA severity or presence (i.e. healthy individual and patients with EOA Spain, vs patients with LOA from Cyprus), but also in other potentially important aspects (e.g., recruitment process, assessment of knee OA, etc.) apart from the actual disease criteria. This also includes the risk of cluster bias as the two counties have significant differences in population, geography, and healthcare system, however, this work was part of a European project involving the recruitment of individuals from two different countries (i.e. Spain and Cyprus). Despite this, we managed to follow the same protocols for the recruitment of patients and the same standard operation processes for blood collection, handling, and storage as well as for the determination of the concentration of serum biomarkers. The patient number of our study was relatively small, while the sample size was not appropriately powered and thus further studies with larger participant numbers and properly powered are needed. The study was also limited by the fact that no control group (i.e., healthy non-obese young individuals) was included. Therefore, a comparison of biomarker and pain levels between healthy controls and our study population was not carried out in our analysis. All patients with established OA had KL scores ranging from 2 to 4 and suffered from clinically relevant advanced knee OA and thus they underwent knee replacement surgery. Thus, the reported biomarker levels represent the bone and cartilage turnover status of patients requiring surgery mainly due to functional/pain limitations and/or OA-induced symptoms. It should also be noted that the majority of patients with established (late) OA (mild OA and severe OA groups) suffered from bilateral knee OA. It has been proposed that the presence of unilateral OA may influence patient-reported physical function impairment more than bilateral OA [70]. Another limitation of our study was that we determined biomarker levels using ELISA, which may introduce both systematic and random errors. Moreover, while successful validation of a biomarker via ELISA depends on several factors including patient selection, collection methods, assay selection/handling, and stability of the marker. Nevertheless, we developed our SOPs for the determination of biomarkers levels via ELISA to minimize potential errors.

In this study, we were only able to measure the concentration of sCOMP, sPIICP, and sHA despite other biomarkers such as PIIANP [39], C-telopeptide of type II collagen (CTX-II) [67, 71], and synovial cytokines [72], and others [19] have been reported. Moreover, there was a statistical limitation because the relationship between each of the three biomarkers and the severity of OA and other OA risk factors was assessed using two tests, linear regression analysis, and Spearman’s Correlation test. We did not use other analysis methods, e.g., multivariate regression analysis, because we did not want to statistically overestimate. Biomarker levels might be influenced by other factors, including age, obesity, gender, and disease stage.

## Conclusions

Despite the aforementioned limitations, the results of this work based on a general population consisting of healthy individuals at high risk of developing OA, patients with early OA as well as patients with established OA, demonstrated that the sPIICP concentration is strongly associated with the severity of knee OA, knee pain both at resting and during walking and OA risk factors. Importantly our study highlights the potential of PIICP as a serum diagnostic biomarker for osteoarthritis and may facilitate diagnosis and monitoring the progression of the disease with relatively high sensitivity and specificity while the cutoff value of PIICP concentration predictive of the progression of OA was estimated. Further studies on the biomarkers that we examined here and the inclusion of additional biomarkers of the bone and cartilage turnover, in larger groups, are needed to make the results of this study more reliable and objective.

## Data Availability

The data used to support the findings of this study will be available with the request to the corresponding authors.

## Abbreviations

AUC: Area Under Curve
BMI: Body mass index
COMP: Cartilage oligomeric matrix protein
EOA: Early osteoarthritis
HA: Hyaluronan
IQR: Interquartile range
K&L: Kellgren and Lawrence
LOA: Late osteoarthritis
OA: Osteoarthritis
PIICP: Procollagen type II C-terminal propeptide
SD: Standard deviation
